# Energy consumption and carbon emission assessment of battery swapping systems for electric motorcycle

**DOI:** 10.1016/j.heliyon.2023.e22887

**Published:** 2023-11-26

**Authors:** Patcharawat Charoen-amornkitt, Kotchakarn Nantasaksiri, Kitchanon Ruangjirakit, Yossapong Laoonual

**Affiliations:** a Electrochemical Energy Storage and Conversion Laboratory, Department of Mechanical Engineering, Faculty of Engineering, King Mongkut's University of Technology Thonburi, Bangkok, Thailand; b Mobility & Vehicle Technology Research Center, King Mongkut's University of Technology Thonburi, Bangkok, Thailand; c Department of Mechanical Engineering, Faculty of Engineering, King Mongkut's University of Technology Thonburi, Bangkok, Thailand

**Keywords:** Battery swapping system, Carbon emissions, Photovoltaic systems, Electric motorcycle

## Abstract

This work investigated different conceptual models for electric motorcycles, which are electric motorcycles with a home charging system, electric motorcycles using a battery swapping system, and electric motorcycles with battery swapping and photovoltaic systems, in four Southeast Asia countries. The current research focused on analyzing the impact of factors such as the number of battery packs in a swapping station, variation in battery swapping demand, the season, and photovoltaic panel size on energy utilization and carbon emissions associated with the entire energy supply chain. The objective of the current study was to evaluate and compare the well-to-wheel emissions using these different conceptual models, considering the varying energy mixes in four countries with a significant prevalence of motorcycle ownership, Thailand, Vietnam, Malaysia, and Indonesia. The results revealed that by using a 3 kW photovoltaic system, the dependency on grid energy can be significantly reduced and thus provide the highest benefits in terms of reduction of fossil fuel use and CO_2_ emissions. Although switching from internal combustion engine motorcycles to electric motorcycles could substantially reduce carbon emissions, it is only feasible when the primary resources used for generating electricity are sufficiently clean or battery swapping stations are equipped with a 3 kW photovoltaic system. In these four countries, electric motorcycles with battery swapping systems could accelerate the transition to a net-zero carbon emission society by reducing CO_2_ emissions by around 2.6–3.0 Mt-CO_2_ per year in the right environment. Prioritizing the decarbonization of power generation should be the primary focus, considering its critical role as a bottleneck within the system. The findings of this research hold significant value for decision-makers and investors who are actively pursuing smart city development and aiming to harness the potential of renewable energy sources.

## 1 Introduction

**Fig. 1:**
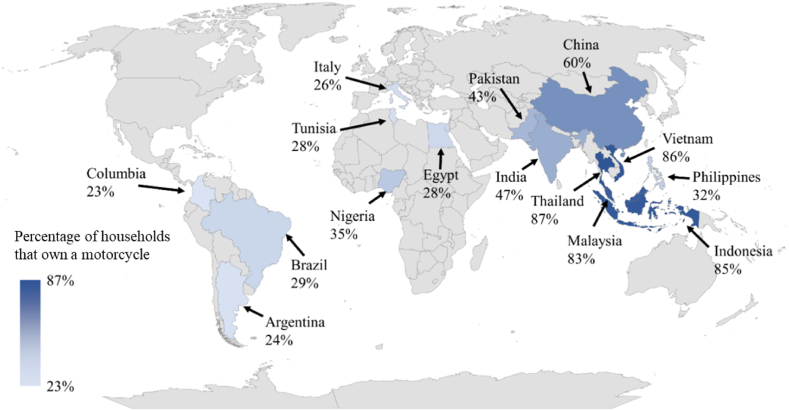
Countries with the highest percentage of households owning motorcycles in 2021 adopted data from [1].

Motorcycles have grown their popularity around the world because of their practicality and low fuel consumption, making them an effective mode of transportation in both large and small cities. As a result, the number of motorcycles in many countries has increased significantly. Of all the motorcycles in the world, approximately 58 % are in the Asia Pacific area and other Asian regions such as Southern and Eastern Asia [1]. Fig. 1 shows the top fifteen countries with the highest percentage of households that own motorcycles. Thailand ranks the highest in this regard, as 87 % of the households in the country own at least one motorcycle. As of now, Thailand has 22.1 million registered motorcycles out of a total of 43.4 million registered vehicles, with just 16,468 and 9023 being battery and hybrid electric motorcycles, respectively [2]. However, excessive use of conventional energy sources, particularly fossil fuels, contributes to global concerns such as energy crises and CO_2_ emissions. Therefore, electrifying the transportation industry has received considerable attention in recent decades because it is a way to solve the energy supply problems and help decarbonize society.

Although electrifying the transportation industry leads to decarbonization, indirect emissions are sensitive to the energy mix. Besides the indirect emissions, some issues that deter potential electric vehicle users are the high cost of energy storage and the long charging times required compared to conventional energy sources. This is due to the limitation of current energy storage and conversion technology. Several studies have been conducted to improve electrochemical energy device performance [[3], [4], [5], [6], [7]]. However, this approach is still ongoing and needs more advanced technologies. Therefore, it may not be effective in accelerating the transition to electric vehicles. As one of the main barriers to switching to electric vehicles is the cost of electric vehicles and their batteries, battery swapping systems are an attractive option. This is due to their rapid replacement of discharged batteries, high flexibility in charging time and power, and the lower upfront cost of electric vehicles. Through this system, an electric vehicle can visit a battery swapping station where a discharged battery can be quickly and easily replaced with a fully charged one [8,9].

The concept of battery swapping has been proposed for several decades. However, due to the lack of interest in industry-wide battery standardization and high investment costs, development of battery swapping has been overlooked. It was not until 2007 when Better Place sparked interest in battery swapping systems. Better Place developed its battery swapping process and signed a memorandum of understanding with Renault-Nissan to build an electric recharge grid. Despite these efforts, market penetration was far lower than originally predicted, leading to the company's bankruptcy [10]. In 2013, Tesla announced its plan and prototype for a battery swapping system but canceled it in 2015 [11]. Recently, Nio launched a business model of selling electrics vehicle without batteries, but the owner has to subscribe to leasing their battery pack [12]. As batteries are the most expensive electric vehicle component, this saves owners large amounts of money on the vehicle's price.

**Fig. 2:**
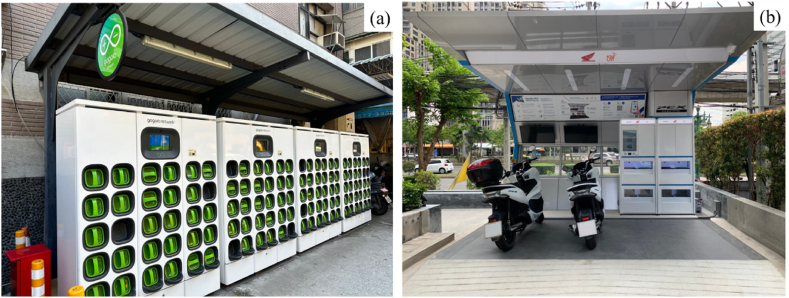
Battery swapping stations in Asia (a) Gogoro network in Taipei City, Taiwan (b) Honda PCX battery swapping station project at KX - Knowledge Exchange for Innovation building, Bangkok, Thailand.

Establishing a battery swapping system for four-wheel vehicles has proven to be challenging due to difficulties in developing complex and fully automated swapping systems as well as the high capital costs involved. However, battery swapping systems for motorcycles and light electric vehicles have gained significant attention in recent years, particularly in Southeast Asia. Motorcycle battery swapping stations typically require a semi-automated swapping system designed for riders to manually swap the battery packs themselves. For motorcycle applications, Honda, Yamaha, Kawasaki and Suzuki announced their collaboration on the development of battery swapping technology back in 2019. This was done to standardize technology and ensure that batteries can work properly across motorcycle brands [13]. In 2021, Honda, KTM, Piaggio, and Yamaha signed an agreement to jointly develop a battery swapping system [14]. Aside from these large companies, several others are operating their battery swapping service in Asia, including Gogoro and Kymco in Taiwan, Swap Energi in Indonesia, Oyika in Singapore, ETRAN, Honda, Swap & Go and Winnonie in Thailand. Fig. 2a and b depicts some battery swapping services provided by Asian companies.

Southeast Asian countries exhibit positive attitudes towards battery swapping technology, as is evident through the presence of service providers and government support. In Thailand, for example, Swap & Go, a business under the PTT Group, has established 22 battery swapping stations covering over 150 km in Bangkok [15]. Their objective is to create the largest EV battery network for two-wheelers in the country, contributing to Thailand's electric mobility transition [16]. Notably, Swap & Go has partnered with Thoresen Thai Agencies Public Company Limited to expand the battery swapping network for electric motorcycles [17]. In 2023, Thailand initiated field testing for a swappable battery platform specifically designed for electric motorcycles, showcasing the country's commitment to innovation and collaboration between public and private sectors [15]. This project addresses a crucial challenge in battery swapping systems by standardizing the battery pack. Thailand, along with Indonesia, Malaysia, and Vietnam, has emerged as a regional leader in electric vehicle production, supported by government incentives and efforts to attract investments and create employment opportunities [18]. Despite limited information on battery swapping policies, the presence of service providers and the government's commitment signify a positive outlook for the technology in Southeast Asia.

There has been research on various aspects of battery swapping systems, including swapping techniques [[19], [20], [21]], optimal locations for battery swapping systems [[22], [23], [24], [25]], energy management [[26], [27], [28], [29]], and integration of renewable energy into battery swapping stations [[30], [31], [32], [33]]. To reduce indirect emissions, integrating renewable energy into the power generation system is important [[34], [35], [36]]. Theoretically, renewable energy can make electric vehicles an environmentally friendly solution. Jordehi et al. [30] studied optimal location of battery swapping stations in a microgrid with micro-pumped hydro-storage, photovoltaic, wind and geothermal units. The results revealed that the difference between the best and worst locations in daily operation cost of the microgrid is approximately 4.9 %. Furthermore, the optimal location also depends on the station's size and maximum charging power. Feng et al. [31] optimized a battery swapping station integrated with a photovoltaic system considering weather and traffic forecasts and speed-variable charging. For the speed variable charging, if there is a battery newly connected into the last charging slot when other battery packs are fully charged, the battery will be charged at a slower rate. However, a constant rate is used at all times for conventional charging. It was found that the speed-variable charging proposed in this study is helpful for peak shaving as it can utilize photovoltaic-generated energy intelligently. Recently, Wang et al. [32] conducted a multi-objective optimization for the charging schedule of battery swapping stations to promote peak shaving by utilizing loads in battery swapping stations. Size optimization of photovoltaic and energy storage in an off-grid system for an application of battery swapping stations was studied by Ban et al. [33]. This study showed the potential of an off-grid system for battery swapping stations as it offered economic benefits, reliability, and avoidance of photovoltaic and energy storage system underutilization.

A notable gap in the aforementioned studies is the lack of consideration regarding the origin of the electricity used for charging electric vehicles, which is a crucial factor to consider. When power is produced from non-renewable energy sources, such as coal or natural gas, charging EVs can still produce significant carbon emissions. As previously highlighted, electric vehicles can only have a positive environmental impact if a substantial proportion of the energy used to produce electricity are obtained from renewable sources. Michaelides [37] evaluated carbon emissions based on a regional mix of electricity generation and well-to-wheel efficiency. The findings revealed that in nations where a large portion of electricity is generated by fossil fuel combustion, switching to electric vehicles may increase CO_2_ emissions. Andersson and Börjesson [38] performed a life cycle assessment of a hybrid electric vehicle, a plug-in hybrid electric vehicle, and a battery-electric vehicle using the 2020 EU-28 electricity mix. A battery electric vehicle's CO_2_ emissions are significantly higher over its lifetime than a hybrid electric vehicle that operates on E85, a mix of 85 % ethanol and 15 % gasoline. Assessment of life cycle environmental impacts and carbon emissions for electric and gasoline vehicles in China was conducted by Yu et al. [39]. As approximately 78 % of electricity in China is generated from coal, electric vehicles have more significant overall environmental consequences than gasoline vehicles. As a result, electrifying automobiles is only the first step toward achieving global net zero greenhouse gas emissions by 2050, as outlined in the 2021 United Nations Climate Change Conference (a.k.a. COP26). Moving away from the heavy dependence on fossil fuels in power generation is necessary. Therefore, integrating electric vehicles with renewable energy resources is essential.

Despite previous research on integration of photovoltaics into battery swapping stations [[30], [31], [32], [33]], the focus has primarily been on optimizing charging schedules, particularly peak shaving. However, it is important to note that charging schedule optimization is not the only crucial factor for photovoltaic-integrated battery swapping systems. Emissions reduction also requires careful investigation. There is currently a lack of studies that analyze the energy consumption of a charging station with integrated photovoltaics and battery swapping, specifically in terms of the capacity represented by the number of charging slots and batteries. Motivated by the problem background surrounding the unique characteristics of battery swapping and its potential impact on mode shift and carbon emissions, this research aims to fill the existing knowledge gap by addressing the following specific unknowns:

1. Does the integration of a battery swapping system offer environmental advantages over the plug-in charging approach?
2. What is the potential scale of climate benefits that can be achieved through implementation of a photovoltaic-integrated battery swapping system?

The reason for this is that operating a swapping station necessitates additional power. If the energy mix has a large proportion of fossil fuels, this possibly leads to higher emissions. Thus, implementing battery swapping systems will not necessarily provide benefits for transitioning to a renewable and sustainable energy society. Second, to the best of our knowledge, the environmental benefits of photovoltaic-integrated battery swapping have never been quantitatively evaluated. Previous studies suggested utilizing a photovoltaic-integrated battery swapping system offers environmental benefits. However, the magnitude of the climate benefits obtainable from such a system needs to be clarified. Therefore, an investigation of the effects of different photovoltaic-integrated battery swapping station designs on energy utilization and well-to-wheel emissions needs to be conducted to answer the aforementioned questions. Understanding the effects of different scenarios where internal combustion engine (ICE) motorcycles, electric motorcycles with home charging systems, electric motorcycles with a battery swapping system, and electric motorcycles with battery swapping and photovoltaic systems are employed is essential for enhancing our understanding of battery swapping systems.

## 2 Objectives

The main objective of the current study is to investigate energy utilization and carbon emission of different designs of battery swapping stations for electric motorcycles that employ photovoltaic systems. Seasonal effects on photovoltaic performance were also considered. Later, the well-to-wheel emissions of various conceptual models with different energy mixes, Thailand, Vietnam, Malaysia, and Indonesia, were evaluated and compared. This research is particularly relevant to sustainable cities and societal development in the Southeast Asia region. As many countries in this region have a large number of motorcycles, the findings of this study could help these nations to decarbonize and transition towards renewable and sustainable energy societies. By understanding the energy utilization and well-to-wheel emissions of different battery swapping station designs, decision-makers and investors can make informed decisions on how to reduce their carbon footprint and accelerate the transition towards a more sustainable future.

## 3 Methodology

### 3.1 Overall procedure

**Fig. 3:**
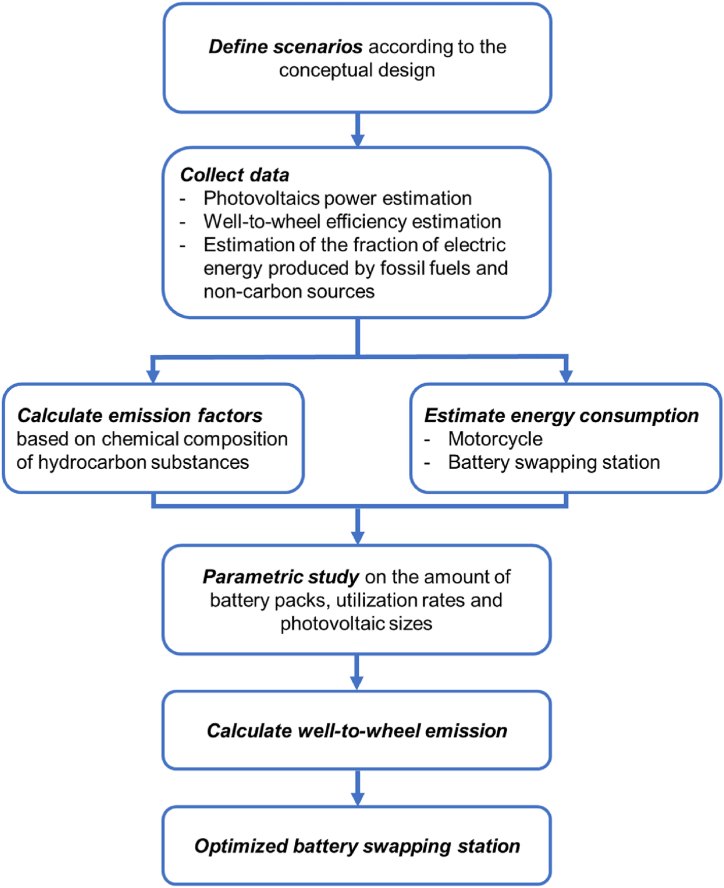
Step-by-step calculation process implemented for the overall procedure.

In this study, the methodology involved conducting simulations and modelling to assess the energy utilization and well-to-wheel emissions of several battery swapping station designs. Various parameters, such as the energy mix of each country, transportation patterns, and specific driving conditions, were incorporated into the models to provide a comprehensive analysis. The methodology employed to study the energy utilization of various battery swapping station designs with photovoltaic systems is summarized in Fig. 3. Subsequent subsections cover defining scenarios and parameters in photovoltaic-integrated battery swapping systems, collecting data, and emission factor and well-to-wheel efficiency calculation.

### 3.2 Defining scenarios and parameters in photovoltaic-integrated battery swapping systems

In this study, to estimate the optimal design for a battery swapping station, the requirements for the number of battery packs in the station, variation of battery swapping demand, the season, and photovoltaic size on energy utilization and well-to-wheel carbon emissions were investigated. The three models considered in this work are electric motorcycles with a home charging system, electric motorcycles using a battery swapping system, and electric motorcycles with battery swapping and photovoltaic systems compared to ICE motorcycles.

**Fig. 4:**
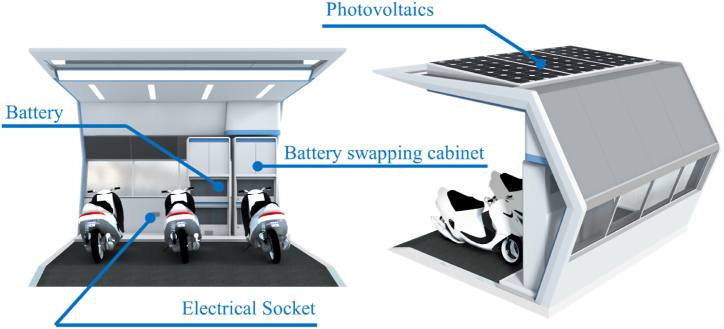
Conceptual design of the battery swapping station [40].

The dimensions of the battery swapping station considered in this study are 4 × 4 × 3 m, capable of parking 3–4 electric motorcycles (see Fig. 4). The system is assumed to provide service from 6:00 to 22:00. Based on the motorcycles available in the market, an electric motorcycle with two battery packs and a total energy capacity of 2 kWh was selected. The driving range of such an electric motorcycle is 40 km. However, an ICE motorcycle can travel around 430 km using 8.1 L of gasoline. Since electric motorcycles are designed to operate with two battery packs, there are always two charging slots available at swapping stations. The energy consumption of the battery swapping station can be categorized into two main types, the energy consumption due to the components of the station and the energy consumption due to the battery swapping demand. Table 1 provides information on the components proposed in this study. The system consists of closed-circuit television (CCTV), linear lightbulbs, a display screen, and other accessories. The CCTV operates 24 h a day, while the lighting is assumed to be run from 18:00 to 06:00 during the night time. The display screen is always on during service time. Other accessories, such as devices for storing and transferring station data, operate 24 h a day.

**Table 1:** Details of equipment considered in the battery swapping system in this study.

Equipment	Consumption (W)	Quantity	Operating hours
CCTV	50	1	00:00–24:00
Linear lightbulbs	50	5	18:00–06:00
Display screen	100	1	00:00–24:00
Other accessories	10	5	00:00–24:00

**Fig. 5:**
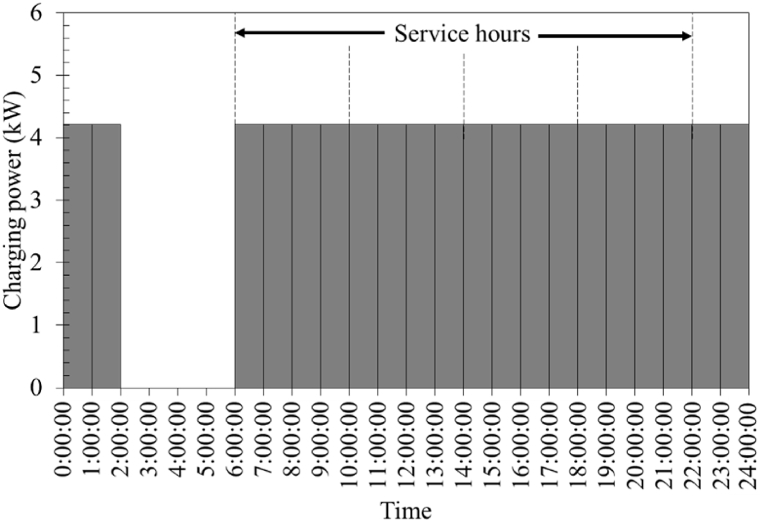
Simulated charging power in the case where the number of battery packs in the station is 32, with a constant utilization rate of 50 % (a station designed for motorcycles traveling a cumulative distance of 1600 km per day). The dashed line indicates the swapping events.

Considering the available space, the number of battery packs in the station was set to 16, 24, and 32 packs to estimate the energy consumption due to the battery swapping demand. The utilization rate of the battery pack in each case is assumed to be approximately 20 %, 50 %, and 80 % at all times. For example, the utilization rate of 50 % for the case where the number of battery packs in the station was 32 packs means 16 packs of the battery will be charged at all times (see Fig. 5). This station is designed for motorcycles cumulatively traveling 1600 km per day. Table 2 summarizes the number of battery packs utilized in different cases. The assumption of constant charging power was used and the charging time was set to 4 h from 0 to 100 % state of charge. Thus, the battery packs will be swapped once every 4 h. It is noteworthy that since consumers would to charge a battery pack faster using a home charging system, the charging time was assumed to be 1 h for electric motorcycles with a home charging system.

**Table 2:** Number of battery packs charged at all time.

The number of battery packs in the station	Utilization rate		
	20 %	50 %	80 %
16	2 (200 km per day)	8 (800 km per day)	12 (1200 km per day)
24	4 (400 km per day)	12 (1200 km per day)	20 (2000 km per day)
32	6 (600 km per day)	16 (1600 km per day)	26 (2600 km per day)

### 3.3 Collecting data

#### 3.3.1 Photovoltaics power estimation

**Fig. 6:**
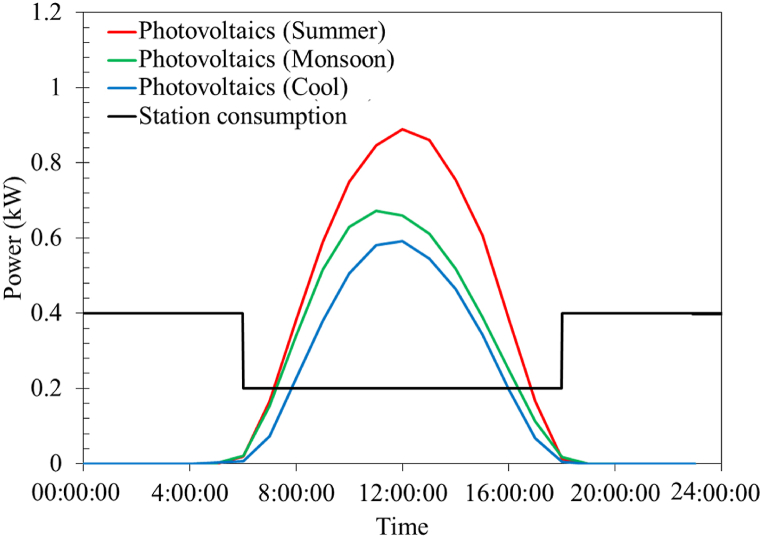
Average photovoltaic-generated power using three solar panels (1 kW) during different seasons in Bangkok, Thailand, and the base power consumption of the battery swapping station considered in the current study.

The hourly solar irradiation (W m^−2^) used in this study was measured from 2012 to 2016 at the Department of Alternative Energy Development and Efficiency [41], a department under Thailand's Ministry of Energy in Bangkok, Thailand. These data will be utilized to investigate the effects of photovoltaics in Thailand, Vietnam, Malaysia, and Indonesia. It is assumed that the hourly solar irradiation of each considered country was similar as all the nations are in Southeast Asia. The weather in this region is hot and humid, with a long monsoon season. There are three official seasons, summer (March to May), monsoon (June to October), and cool (November to February). The intensity of solar irradiation is different in each season. Hence, the hourly solar irradiation data were classified into three seasons to account for the effects of seasons on photovoltaic power generation. The solar panel dimensions were 1960 × 990 mm. This work considers sets of three and eight panels with an energy conversion efficiency of 20 %. The three panels will generate approximately 1 kW of electricity and the available space on the station's roof is sufficient for only eight panels (3 kW). Assuming no shading of the photovoltaic panels by other buildings, the hourly photovoltaic power can then be estimated directly from the solar irradiation data. Fig. 6 depicts the hourly photovoltaic power of three photovoltaic panels in different seasons, together with the station's energy consumption due to its components (presented in Table 1). As expected, during the summer, the photovoltaic system can generate the highest power and a decrease of around 33 % is observed during the cool season.

#### 3.3.2 Well-to-wheel efficiency estimation

To account for the emissions during the entire chain of processes that power the wheels of motorcycles, starting with the primary energy sources, the processes considered in this study are fuel transportation to the power plant, fuel conversion to electricity, electric power transmission from the power plant to the consumer, battery charging, and power supply from the tank to the wheels.

The efficiency of fuel transportation to a power plant refers to the percentage of the energy contained in the fuel that remains before it is converted into electricity at the power plant. There are several factors that can impact the efficiency of fuel transportation, including the mode of transportation (*e.g.*, truck, train, pipeline), the distance the fuel needs to be transported, and the energy required to transport the fuel (*e.g.*, fuel consumption of the vehicles or the energy required to pump the fuel through a pipeline). However, no fuel transportation is required for other energy resources like wind and solar energy.

The efficiency of the fuel conversion to electricity refers to the percentage of the energy contained in the fuel that is converted into electricity. The overall efficiency of the fuel conversion process can be influenced by a number of factors, including the type of fuel being used, the efficiency of the equipment used to convert the fuel into electricity, and the ambient conditions in which the conversion takes place. The efficiency of fuel conversion is typically measured in terms of the “energy conversion efficiency,” which is defined as the ratio of the energy output (in the form of electricity) to the energy input (in the form of fuel). For example, if a power plant has an energy conversion efficiency of 40 %, it means that 40 % of the energy contained in the fuel is converted into electricity, while the remaining 60 % is lost as waste heat. The efficiency of fuel conversion to electricity process varies depending on the type of energy resources used to produce the electric power. For instance, the energy conversion efficiencies of photovoltaic, nuclear, wind turbine, gas turbine, coal-fired, and hydroelectric power plants are 18 %, 30 %, 30 %, 35 %, 40 %, and 75 %, respectively [42,43]. There are a number of ways to improve the efficiency of the fuel conversion process, including the use of advanced technologies and equipment, optimizing operating conditions, and adopting best practices in energy management. Improving the efficiency of fuel conversion can help to reduce the carbon footprint of power generation and lower the cost of electricity.

After electricity is generated at a power plant, it must be transmitted to the users. In this process, the losses in the transmission lines and transformers are considered. There are several types of losses that can occur in transmission lines and transformers:

1. Ohmic losses: These are losses that occur due to the resistance of the transmission lines and transformers. As electricity flows through these components, it encounters resistance, which causes some of the energy to be dissipated as heat.
2. Eddy current losses: These losses occur in transformers due to the induced currents that flow in the core of the transformer. These currents create a resistance, which causes energy to be lost as heat.
3. Hysteresis losses: These losses occur in transformers due to magnetization and demagnetization of the core material as the transformer operates. This process also generates heat, which results in energy losses.
4. Stray losses: These losses occur due to the leakage of magnetic fields around the transformer, which can cause energy to be lost as heat.

Typically, the value of transmission efficiency is 90–95 % for developed countries, while it is 85 % for developing countries [44,45].

As batteries possess an internal resistance due to electrode structures, energy dissipation during charging is inevitable. If a 350 V battery with a capacity of 250 Ah (87.5 kWh) and a total resistance of 0.2 Ω is subjected to a charge rate of 2C (500 A), the charging loss will be approximately 28.5 % without considering the balance of the plant. However, the loss is not apparent in the case of a motorcycle because of the much lower charging current. In this study with the 20S7P battery arrangement, the resistance of the battery pack is assumed to be approximately 0.1 Ω. Taking the efficiency of the power electronic system into account, the charging efficiency is found to be around 95 % and 90 % for 1C and 0.25C charging, respectively.

There is some energy loss while supplying power from the tank to the wheels. “Tank-to-wheel” efficiency refers to the efficiency of a vehicle in converting the energy stored in its fuel into useful work (*i.e.*, moving the vehicle). This process includes battery discharge, traction motors, and gear box efficiency. In general, ICE vehicles have lower tank-to-wheel efficiencies compared to electric vehicles. This is because electric vehicles do not have energy losses associated with the combustion process and can convert a higher percentage of the stored energy in their batteries into useful work. For electric vehicles, the combined efficiency is in the range of 80–90 % [37]. However, the typical efficiency of ICE vehicles is around 16–20 % [37]. It is notable that although the tank-to-wheel efficiency of ICE vehicles is significantly lower, the losses due to fuel conversion to electricity, electric power transmission from the power plant to the consumer, and battery charging are not considered. This could result in more energy required for electric vehicles when the well-to-wheel processes are considered. Table 3 summarizes the efficiencies used for estimating the well-to-wheel efficiency of ICE and electric motorcycles in this study.

**Table 3:** Efficiencies for ICE and electric motorcycles.

Scenarios	Efficiency				
	$\eta_{ft}$	$\eta_{con}$	$\eta_{tr}$	$\eta_{ch}$	$\eta_{ttw}$
ICE motorcycles	0.8	–	–	–	0.2
Electric motorcycles	0.8	0.2–0.75	0.85	0.90–0.95	0.8

#### 3.3.3 Estimation of the fraction of electric energy produced by fossil fuels and non-carbon sources

One of the most appealing aspects of electric vehicles is that they do not emit any pollutants. However, this is true only when only the vehicles are considered, as electric vehicles do not have tailpipe emissions. The power generation process at the power plants still produces significant pollution if fossil fuels are used. As discussed earlier, electrifying vehicles is just the first step toward achieving global net zero greenhouse gas emissions because it allows us to easily use renewable energy resources. If the electricity supplied to electric vehicles is generated using energy sources such as nuclear, hydroelectric, solar, wind, biomass, and waste products, significant CO_2_ avoidance is accomplished by switching to electric vehicles. However, if fossil fuels are used in a large portion for producing electricity, replacing ICE vehicles with electric vehicles results in a net increase in CO_2_ emissions [37].

As of 2021, in Thailand, natural gas accounted for 64.1 % of Thailand's electrical production, followed by coal, hydroelectric, and oil. Renewable energy accounted for only 10 % of the total energy production, around 9053 MW. Biomass power plants were the largest power plants that did not use fossil fuels, accounting for more than 29 % of the total installed capacity. This is followed by solar energy and large hydropower plants, approximately 24 % each. However, the power generation from renewable energy at this moment is only 53 % of the target set by Thailand's ministry of energy, which is 16,778 MW from a renewable-based power supply by 2036 [46].

Vietnam's energy mix is dominated by fossil fuels, with coal and natural gas accounting for most of the country's energy production. According to the International Energy Agency [47], in 2020, coal accounted for around 49.6 % of Vietnam's total primary energy supply, followed by natural gas at 14.2 %. Renewable energy sources, including hydropower, solar, and wind, comprised the second-largest share of Vietnam's energy mix at around 35.4 %.

With coal accounting for over 62 % of the nation's electricity production, Indonesia depends mainly on fossil fuels [48]. The Indonesian government is working to boost the use of renewable energy sources, although most new power plants still rely heavily on fossil fuels. With just around 18.9 % of the nation's total electricity coming from renewable sources, Indonesia's development in renewable electricity generation is noticeably slower than that of coal.

For Malaysia, the International Energy Agency [49] estimates that in 2019, coal and natural gas made up around 83.3 % of Malaysia's total primary energy supply, with coal use of 47 % and natural gas of 39.3 %. Renewable energy comprises around 16.2 % of Malaysia's energy mix.

Table 4 shows the fraction of electric energy produced by fossil fuels and non-carbon energy resources in the four countries examined in the current study. Since fuels have varying chemical composition, the carbon emissions from each fuel type is also different. This work calculated the carbon emissions emitted during power generation from fuel with assumed chemical compositions (detailed in Section 3.4 below). However, the obtained emission factors did not represent the total process emissions. This is because the losses due to transportation of the primary energy resources, electric power transmission from the power plant to the consumer, and battery charging still need to be evaluated. The emissions during the processes listed in Table 3 are required evaluate the total emissions. It is notable that the fraction of electric energy produced outside the country is not included in Table 4. Combining the fraction of electric energy produced with their emission factors and the well-to-wheel efficiency, the well-to-wheel emissions of each scenario can be estimated.

**Table 4:** The fraction and emission factor of electric energy produced by fossil fuels and non-carbon sources in four countries with the highest percentage of households with motorcycles.

Type of fuels	Chemical formula	Thailand	Vietnam	Indonesia	Malaysia	Emission factor (kgCO_2_/kWh)
		Fraction of electric energy produced [46]	Fraction of electric energy produced [47]	Fraction of electric energy produced [48]	Fraction of electric energy produced [49]	
Lignite coal	C_39_H_35_O_10_NS	0.205	0.496	0.620	0.470	0.91
Natural gas	95 % CH₄, 4 % C₂H₆, 1 % C₃H₈	0.641	0.145	0.165	0.363	0.52
Petroleum (Power plant)	C₈H₁₈	0.004	0.004	0.027	0.005	0.60
Non-carbon	–	0.150	0.354	0.189	0.162	–
Petroleum (Motorcycle)	C₈H₁₈	–	–	–	–	1.21

### 3.4 Emission factor and well-to-wheel efficiency calculation

The emission factor employed in this study serves as a quantitative measure to assess the greenhouse gas emissions released per unit of electricity generated. Specifically focusing on carbon dioxide emissions, it provides a valuable tool for policymakers, researchers, and businesses to evaluate the environmental impact of specific activities and guide efforts for emission reduction through targeted interventions and policy measures. Typically, emission factors for electricity generation using different fuels can be obtained from the existing literature. In this study, instead of relying solely on published emission factors, our approach involved calculating the carbon emissions during power generation based on assumed chemical compositions. The ratio of carbon dioxide emissions per unit mass, taking into consideration the specific chemical composition of the fuel being utilized, can be written as shown in Eq. (1).

(1) $$w_{CO_{2}}=\frac{1,000m_{CO_{2}}}{m_{i}}$$

where $m_{CO_{2}}$ is the mass of CO_2_ generated after combustion (g/mol) and $m_{i}$ is the total mass of each assumed chemical substance i (g/mol), while 1000 is used to convert the ratio into the units of grams of CO_2_ per kilogram of chemical substance i. Assuming stoichiometric combustion, all of the carbon present in the fuel will be converted into CO_2_. Therefore, knowing the chemical composition of the fuel, it becomes possible to estimate the mass of CO_2_ produced based on the mass of carbon in the fuel.

In conjunction with the heating value, which represents the amount of heat energy released by a fuel during complete combustion, it is possible to estimate the CO_2_ emissions per unit of energy input. In this context, Eq. (2) expresses the formula to calculate the CO_2_ emission ratio ${EF}_{CO_{2,input}}$.

(2) $${EF}_{CO_{2,input}}=\frac{w_{CO_{2}}}{{HV}_{i}}$$

where ${HV}_{i}$ is the heating value of chemical substance i (kWh/kg). To estimate CO_2_ emissions per unit of electricity output ${EF}_{CO_{2,output}}$, one can use the efficiency of energy conversion systems, which can be expressed as written in Eq. (3).

(3) $${EF}_{CO_{2,output}}=\frac{{EF}_{CO_{2,input}}}{\eta_{j}}$$

where $\eta_{j}$ is the efficiency of energy conversion system j.

Taking all the processes from well to wheel into account, the well-to-wheel efficiency can be expressed by Eq. (4).

(4) $$\eta_{wtw}=\eta_{ft}\eta_{con}\eta_{tr}\eta_{ch}\eta_{ttw}$$

where $\eta_{ft}$ is the fuel transportation efficiency, $\eta_{con}$ is the energy conversion efficiency, $\eta_{tr}$ is the transmission efficiency, $\eta_{ch}$ is the charging efficiency, and $\eta_{ttw}$ is the tank-to-wheel efficiency.

## 4 Results and discussion

### 4.1 Photovoltaics-integrated battery swapping systems: impact on grid energy utilization

**Fig. 7:**
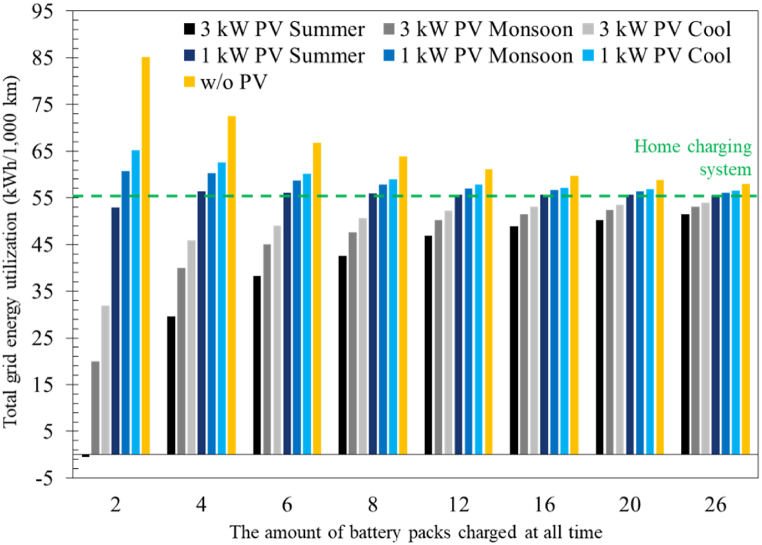
Total grid energy utilization under different scenarios.

The impacts of battery swapping and photovoltaic systems on grid energy utilization were investigated. Fig. 7 depicts the energy consumption per 1000 km distance travelled for motorcycles with different scenarios involving various photovoltaic sizes, station utilization rates, and charging systems. The results revealed that if a battery swapping system was utilized, a photovoltaic system was necessary if less grid energy is to be consumed compared to a home charging system. This is because even though the swapping system could provide a higher charging efficiency (95 %) compared to a home charging system (90 %), additional energy was required to operate the battery swapping station. As the station utilization rate is increased, the energy consumption decreased since less energy is required per distance travelled to operate the station. When a 1 kW photovoltaic system was integrated into the station, the photovoltaic-generated power can significantly reduce the proportion of grid energy required. Generally, this was still insufficient to reduce the grid energy utilization to a level lower than a home charging system.

However, when a 3 kW photovoltaic system was implemented, the grid energy utilization was significantly lower than that of a home charging system, especially when the station utilization rate was low. The total grid energy utilization can even be negative when a 3 kW photovoltaic system is utilized in summer (−0.35 kWh per 1000 km). However, the grid energy consumption increased as the station utilization rate increased. This is expected since as the energy demand is rising due to increased utilization, photovoltaic-generated power is limited. Nonetheless, it is clear that if the goal is to implement a battery swapping system to assist the transition to a sustainable energy society, integrating a photovoltaic system into the battery swapping system is mandatory. The total grid energy utilization is the energy input for operating a motorcycle to travel 1000 km. Based on the ICE motorcycle specification in this study, considering the gasoline energy density of 8.9 kWh/l, the energy input would be equivalent to 167.7 kWh per 1000 km. Even though it is a large number and is far higher than that of electric motorcycles, this is unsurprising as the tank-to-wheel efficiency of an ICE is significantly lower. Nonetheless, the numbers are incomparable since the efficiencies from well-to-tank were not considered for the electric motorcycle cases.

**Fig. 8:**
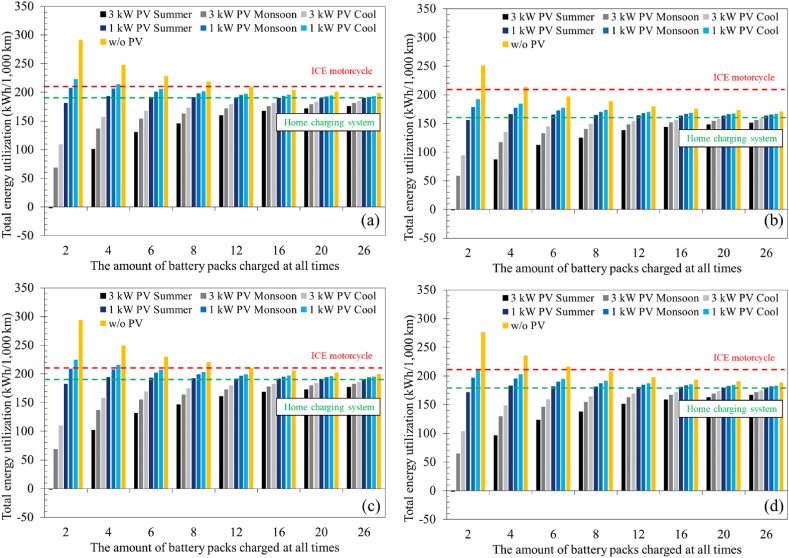
Comparison of total energy utilization considering the energy mix of (a) Thailand, (b) Vietnam, (c) Indonesia, and (d) Malaysia and the efficiency of the entire chain of processes from well to wheels.

Fig. 8a–d shows the total energy utilization per 1000 km considering the energy mix and the efficiency of the entire chain of processes that powers the wheels of motorcycles. This analysis starts with the primary energy sources in Thailand, Vietnam, Indonesia, and Malaysia. The results revealed that the total energy utilization differed depending on the energy mix of each country, although they shared a similar trend. For Vietnam, in Fig. 8b, the energy use is more efficient as approximately 30 % of electricity was generated by hydropower plants. Hydropower plants can achieve an energy conversion efficiency of 75 %, while only a 20 % energy conversion can be obtained through photovoltaics. This resulted in the significantly lower use of primary energy resources. In the case of ICE motorcycles, energy consumption was estimated at around 210 kWh per 1000 km of distance travelled. In all four nations in the current study, electric motorcycles with a home charging scheme provided significantly lower energy utilization than ICE motorcycles. It is notable that considering the primary energy sources, the energy utilization of ICE motorcycles was less than the case when electric motorcycles were utilized in a battery swapping system with no integration of photovoltaics and a low utilization rate (a motorcycle comes to swap its battery pack at a station once every 4 h). This is because some electricity is required to operate the battery swapping station. However, overall, the results suggested that regardless of the implementation, promoting the switch to electric motorcycles would help reduce a significant portion of energy use.

**Fig. 9:**
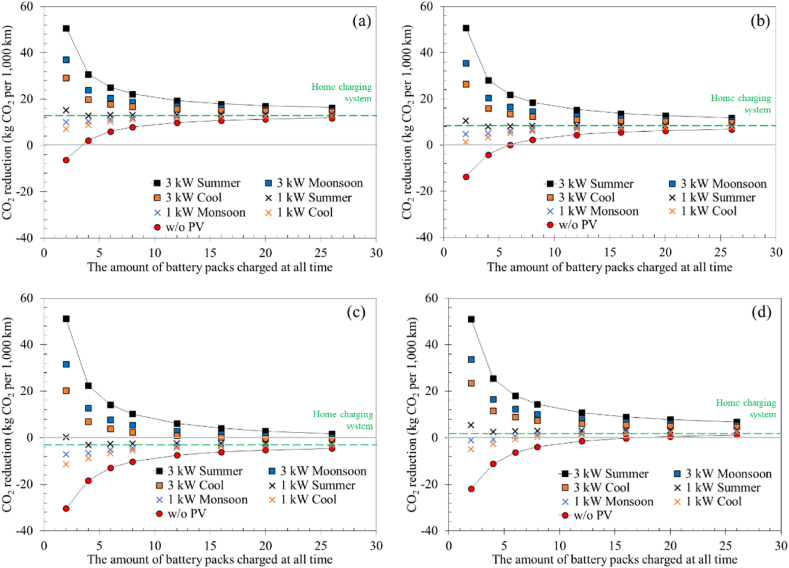
CO_2_ reduction comparing the case of an ICE motorcycle considering the energy mix of (a) Thailand, (b) Vietnam, (c) Indonesia, and (d) Malaysia.

Fig. 9a–d shows the CO_2_ reduction from the switch from ICE motorcycles to electric motorcycles using different implementation approaches. For Thailand, the results revealed a positive impact, as the CO_2_ emissions were reduced in all cases except the one without integration of photovoltaics and a low utilization rate. Based on the calculations in this study, where the gasoline is considered to be octane, the CO_2_ emissions of an ICE motorcycle was 50.6 kg-CO_2_ per 1000 km. It can be clearly seen that, as the station utilization rate increased, the CO_2_ reduction for electric motorcycles with battery swapping systems converged to values of around 14, 9.2, −1.6, and 3.8 kg- CO_2_ per 1000 km for Thailand, Vietnam, Indonesia, and Malaysia, respectively. For Thailand and Vietnam, this number is slightly higher than the case where ICE motorcycles were utilized, showing great benefit of implementing battery swapping systems. However, for Malaysia, the reduction was much less significant, while utilizing electric motorcycles can negatively impact the environment in Indonesia. This is because, in Indonesia, the fraction of electric energy produced from coal was around 62%, which is higher than the other countries. Consequently, electric motorcycles with a battery swapping station integrated with 1 kW photovoltaics or less cannot provide environmental benefits in all scenarios. It should be noted that although Vietnam utilizes much hydropower (30 % of generated electricity), 49.6 % of electrical energy was derived utilizing coal, giving Vietnam less benefit than Thailand. This highlights that the higher emission factor of coal (0.91 kg-CO_2_/kWh as compared to 0.52 and 0.60 kg-CO_2_/kWh for natural gas and petroleum, respectively) indicates that the share of the electricity generated from coal should be minimized. These findings align with previous studies [[37], [38], [39]] that have shown in countries where a significant proportion of electricity is generated through the combustion of fossil fuels, transitioning to electric vehicles could potentially lead to an increase in CO_2_ emissions.

Although the battery swapping system showed potentially great environmental benefits, it is noteworthy that if the utilization rate of a station is low, it is vital to integrate the photovoltaic system to reduce its dependency on grid energy. Otherwise, charging electric motorcycles at home would provide greater benefit. However, if the utilization rate is high, there is no significant difference between integrating and not integrating photovoltaics. This suggests that the environmental benefits of switching to electric motorcycles are substantial only if the emissions from producing electricity are sufficiently low. As space in the station is limited, integrating a battery swapping station into a microgrid should be the best approach for implementing a battery swapping system to assist in transitioning to sustainable societies. Assuming that a motorcycle travelled an average of 10,000 km, with a total of 21.8 million motorcycles in Thailand, if half of them were converted into electric motorcycles, the CO_2_ emission from motorcycles could be reduced by approximately 2.6–3.0 Mt-CO_2_ per year. However, Indonesia is a great example of electric vehicles hindering the transition to a renewable and sustainable energy society. With roughly 120 million motorcycles in Indonesia, considering the abovementioned scenario, approximately 1.0 Mt-CO_2_ would be added into the atmosphere compared to the ICE motorcycles.

However, it is important to acknowledge that integrating renewable systems into existing grid infrastructure faces the challenge of intermittency. Unlike conventional power generation methods, renewable sources, such as solar and wind, can experience fluctuations, resulting in an inconsistent and unpredictable power output. Therefore, caution should be exercised when considering integration of battery swapping stations into microgrids to avoid potential issues with grid stability. To address the intermittency challenge, effective energy storage systems, such as batteries, are typically implemented. These storage solutions can capture surplus energy generated during peak production periods and release it during periods of low production, ensuring a more stable power supply to the grid. In this context, the batteries housed within battery swapping stations can play a role in mitigating fluctuations. In the context of this study, an excess of electricity will occur only when the number of battery packs being charged falls below eight for a 3 kW photovoltaic system or two for a 1 kW photovoltaic system. In such cases, the energy demand of the battery swapping station is sufficiently high, and therefore, there will be no concerns regarding fluctuations since all the electricity generated by the photovoltaic system is consumed within the station. Furthermore, another approach to address the intermittency challenge involves diversifying renewable sources by combining solar, wind, hydro, and geothermal energy. This combination helps balance out fluctuations in power generation, reducing the impact of intermittent supply on the grid. Additionally, promoting flexible grid operations and implementing demand response programs can optimize the utilization of renewable energy. By incentivizing consumers to adjust their electricity consumption during times of high renewable energy generation and low demand, alignment between electricity use and renewable energy availability can be improved. It is crucial to emphasize that achieving a balance between decarbonizing power generation and maintaining grid stability is a complex task. It requires careful consideration of regional factors, grid characteristics, and technological advancements. Collaboration among policymakers, energy companies, and grid operators is essential to effectively address these challenges and ensure a smooth transition toward a decarbonized and stable energy system.

### 4.2 Policy implications of battery swapping systems for electric motorcycles

The lack of specific details regarding battery swapping policies in Southeast Asian countries accentuates the significance of the findings presented in this study for the advancement of sustainable cities and societies, particularly in regions such as Southeast Asia, where there is a substantial number of motorcycles. The implications can be summarized as follows:

1. The transition from ICE motorcycles to electric motorcycles holds the potential to substantially reduce CO_2_ emissions, provided that the energy mix is not heavily reliant on coal. However, it is essential to acknowledge that electrification of vehicles alone may not be adequate to achieve significant reductions in CO_2_ emissions.
2. In order to attain net zero carbon emissions, it is imperative for governments to actively support decarbonization of power generation and implementation of photovoltaic-integrated battery swapping systems. This approach offers benefits such as reducing grid energy dependency and CO_2_ emissions.

To support adoption of battery swapping systems, governments can implement a range of policies aimed at incentivizing and promoting their use. These policies include providing financial incentives such as grants, tax credits, or subsidies to battery swapping service providers that incorporate photovoltaics into their systems. This not only reduces the initial investment costs for establishing battery swapping stations but also decreases grid energy dependency. Alternatively, governments have the choice to either make independent investments or create business opportunities for the private sector in the development of battery swapping infrastructure, akin to the provision of train or bus services. In this case, it is advisable for the government-operated stations to integrate photovoltaic systems to further reduce grid dependency. Such initiatives would expand the network of swapping stations, enhancing accessibility for electric motorcycle owners. Governments should also establish regulations and standards to ensure the safety, interoperability, and quality of battery swapping services. Additionally, to foster sustainable cities and societies, regulations can be introduced requiring battery swapping stations to integrate photovoltaic or wind turbine systems. This comprehensive approach promotes the utilization of renewable energy sources and aligns with the objective of reducing CO_2_ emissions. Furthermore, governments can support battery swapping systems by procuring electric vehicles compatible with battery swapping systems for their own fleets, such as police motorcycles. By demonstrating their commitment to this technology, governments can stimulate demand and cultivate a viable market for battery swapping services. In conclusion, the adoption of battery swapping systems can be facilitated through a combination of financial incentives, infrastructure development, regulatory frameworks, research and development funding, public procurement, and awareness campaigns. These measures, when implemented effectively, contribute to the realization of sustainable cities and societies.

Although the photovoltaic-integrated battery swapping system could play an essential role as it benefits the reduction of grid energy dependency and CO_2_ emission, the benefits decreased as the demand for battery packs increased. This created two essential issues regarding the battery swapping system. First, it is unclear if the integration of the photovoltaic system will be economically justified. The extent of government support for the photovoltaic-integrated battery swapping system depends on the balance between its benefits in terms of reduced grid energy dependency, CO_2_ emissions and the associated investment costs. Therefore, further exploration is required to determine the tradeoffs between these advantages, enabling the government to make informed decisions regarding the level of support needed. Second, in this study, the variable load from battery charging is assumed to be constant throughout the day. However, in actual circumstances, the demand for battery packs is probably time-dependent. The demand could be high in the morning before working hours, at noon, and after working hours. Hence, the load could be variable. Consequently, this leads to an issue related to the balance of the charging schedule. Nonetheless, as the battery swapping system also showed great potential in peak shaving, it should be a way forward in transitioning to a renewable and sustainable energy society while developing more advanced energy storage technologies. Therefore, a further study from the economic perspective of photovoltaic-integrated battery swapping systems considering time-dependent demand should be carried out and is ongoing in our research group. Although this work could be potentially of interest to countries with a high percentage of a household that own motorcycles, this study has broader implications beyond the motorcycle industry. The conceptual idea can be applied to larger systems such as battery swapping systems for light-duty vehicles, which has gained significant attention recently.

## 5 Conclusions

To introduce battery swapping systems extensively, it is vital to consider their impacts on fossil fuel dependency and environmental benefits. In this study, an investigation of the different scenarios and designs of the photovoltaic-integrated battery swapping station was successfully done with various energy mixes. A station's energy consumption was estimated based on different battery utilization rates. The hourly solar irradiation was carefully collected from 2012 to 2016, and the data were averaged over each season to predict reliable photovoltaic-generated power. To provide a whole perspective of energy utilization and carbon emissions, the well-to-wheel efficiency was considered and the emission factors were carefully calculated. The results reveal that a battery swapping station with 3 kW photovoltaics always consumed less grid energy than home charging. However, a system with 1 kW photovoltaics cannot provide benefit for reduction of grid energy dependency and may even consume more grid energy. This occurs since additional energy is required to operate the swapping stations. A battery swapping system must either possess large photovoltaic power or a high utilization rate to be comparable to a home charging system. In terms of CO_2_ emissions reduction, utilizing electric motorcycles was better compared to ICE motorcycles if the primary resources used for generating electricity were sufficiently clean. The results of this study provide information about the energy consumption and CO_2_ emissions reduction of battery swapping systems. It is important for policymakers in the energy sector to understand the benefits and limitations of battery swapping systems. Such understanding will enable them to make informed decisions and formulate policies that can support such systems with confidence, ultimately leading to achievement of a sustainable society. The obtained results can be further used to site swapping stations with sufficiently high utilization rates so that the private section does not need to further invest in photovoltaic systems.

## Data availability statement

Data will be made available on request.

## CRediT authorship contribution statement

**Patcharawat Charoen-amornkitt:** Conceptualization, Formal analysis, Funding acquisition, Investigation, Methodology, Software, Visualization, Writing – original draft, Writing – review & editing. **Kotchakarn Nantasaksiri:** Conceptualization, Formal analysis, Investigation, Methodology, Software, Visualization, Writing – original draft, Writing – review & editing. **Kitchanon Ruangjirakit:** Data curation, Formal analysis. **Yossapong Laoonual:** Formal analysis, Funding acquisition, Conceptualization, Resources, Supervision, Writing - review & editing.

## Declaration of competing interest

The authors declare that they have no known competing financial interests or personal relationships that could have appeared to influence the work reported in this paper.
